# Sequence variants in genes causing nonsyndromic hearing loss in a Pakistani cohort

**DOI:** 10.1002/mgg3.917

**Published:** 2019-08-06

**Authors:** Amjad Khan, Shirui Han, Rongrong Wang, Muhammad Ansar, Wasim Ahmad, Xue Zhang

**Affiliations:** ^1^ McKusick‐Zhang Center for Genetic Medicine, Institute of Basic Medical Sciences, School of Basic Medicine, Peking Union Medical College Chinese Academy of Medical Sciences Beijing China; ^2^ The Research Center for Medical Genomics China Medical University Shenyang China; ^3^ Department of Developmental Medicine, King Abdullah International Medical Research Center (KAIMRC) King Saud Bin Abdulaziz University for Health Sciences, Ministry of National Guard‐Health Affairs (MNGHA) Riyadh Saudi Arabia; ^4^ Department of Biochemistry, Faculty of Biological Sciences Quaid‐i‐Azam University Islamabad Pakistan

**Keywords:** *GJB2*, nonsyndromic hearing loss (NSHL), Pakistani Cohort, Targeted Next Generation Sequencing

## Abstract

**Background:**

Hearing loss or hearing impairment is a clinically and genetically heterogeneous disorder. More than 117 genes were discovered to date in hereditary, nonsyndromic hearing loss (NSHL). Identifying novel gene variants and their frequency in specific populations is valuable for public health and potentially for genetic screening of NSHL.

**Aims:**

To identify the gene variants underlying NSHL in a Pakistani cohort.

**Methods and Results:**

A cohort of 40 school‐aged children with NSHL was initially screened for variants in *GJB2,* the gene with the highest incidence of variants in other populations with NSHL. We found known homozygous as well as compound heterozygous *GJB* variants in 15 individuals. Next, we used targeted next generation sequencing (TNGS) for the remaining 25 individuals and identified 20 different variants in 14 genes (*SLC26A4, KCNQ4, MYO7A, MYO15A, TMPRSS3, ESPN, TMC1, GIPC3, LHFPL5, WFS1, DFNB59, GRXCR1, ESRRB,* and *LRTOMT*).

**Conclusions:**

We described common and novel variants in 15 genes in a Pakistani cohort of NSHL.

To the Editor:

Hearing loss is a clinically and genetically heterogeneous disorder and is the most common human disability (Shrivastava, Shrivastava, & Ramasamy, 2016). Both environmental and genetic factors were shown in the pathogenesis of hearing loss such as iodine deficient diet, infections, ototoxic drug treatment, or gene variants (http://hereditaryhearingloss.org/) (Morton & Nance, 2016; Shrivastava et al., 2016; Wonkam et al., 2013). Nonsyndromic hearing loss (NSHL) can be inherited as an autosomal recessive (AR), autosomal dominant (AD), X‐linked (dominant or recessive), or mitochondrial trait (Liu et al., 2005; Snoeckx et al., 2005; Wang, Han, Khan, & Zhang, 2017). Identifying novel sequence variants in populations provide valuable information for public health, particularly for genetic screening of the NSHL.

In our study, we recruited a total of 40 unrelated individuals affected by NSHL from Bannu and Kohat districts in Khyber Pakhtunkhwa (KPK) province, Pakistan. A clinical questionnaire was used to collect medical history and rule out the history of other diseases potentially affecting hearing and environmental factors such as antibiotic use, excessive noise exposure, and infection that could cause hearing loss. Detailed physical examination did not reveal other abnormal findings than hearing loss. Palpable goiters and other dysmorphic features were not observed in any of the participants with hearing loss; their hearing was ranging from severely impaired to deaf, listed in Figure 1 and Table 1. All of the probands had bilateral, prelingual hearing loss consistent with a congenital origin. There was no previous family history of hearing loss in the participants. The study design and protocol were approved by the Institutional Review Board of (IRB) Quaid‐i‐Azam University Islamabad Pakistan, the Ethical Review Committee (ERC) of Peking Union Medical College (Beijing, China), and China Medical University (Shenyang, China). Written informed consents to conduct and publish the study were obtained from all participating individuals and their parents. All 40 individuals were screened by polymerase chain reaction (PCR) and Sanger sequencing for variants in *GJB2* (gap junction protein beta 2; NM_004004.5) gene which is the most commonly mutated gene in recessive NSHL (Liu et al., 2005; Snoeckx et al., 2005). We found known homozygous as well as compound heterozygous variants in 15 individuals. Four homozygous patients had *GJB2*; c. 370C > T; p.Gln124*, six had *GJB2;* c.35delG; p.Gly12Valfs*2, three compound heterozygotes had *GJB2;* c.[71G > A];c.[231G > A]; p.[p.Trp24*];p.[Trp77*], and two had *GJB2;* c.[−23 + 1G>A]; c.[231G > A]; p.[N/A]; p.[Trp77*] variants respectively (Table 1). The 25 individuals, who were negative for *GJB2* gene variants, were screened by targeted next generation sequencing (TNGS) technology using the Ion Torrent platform for a panel of 63 genes implicated in hearing loss as described previously (Wang et al., 2017). We identified 20 variants in 14 genes (*SLC26A4, KCNQ4, MYO15A, TMPRSS3, ESPN, TMC1, GIPC3, LHFPL5, WFS1, DFNB59, GRXCR1, ESRRB,* and *LRTOMT*). Ten were novel such as two homozygous missense variant in *MYO15A* c.[9518‐2A > G];p.[N/A], c.3944G > A;p.Gly1315Glu*,* one novel variant c.199T > G; p.Tyr67Asp in *LHFPL5* in two unrelated individuals, one novel variant c.2519G > A; p.Trp840* in *ESPN*, one in *KCNQ4* c.1288G > A; p.Glu430Lys, one in *ESRRB* c521G > A; p.Arg174His, one in *DFNB59* c.147T > A; p.Tyr49*, one in *GIPC3* c.759C > G; p.Ser253Arg, one in *TMC1* c.662A > G; p.Tyr221Cys, and one novel variant in *LRTOMT* c.154C > T; p.Arg52Trp (Figure 1, Table 1). The functional effect of the variants was predicted using the in silico tools including Polyphen2 (http://genetics.bwh.harvard.edu/pph2/), Sorting Intolerant from Tolerant (SIFT) (http://sift.jcvi.org/), PROVEAN (http://www.provean.jcvi.org) Mutation Taster (http://www.mutationtaster.org/), Human Splicing Finder (HSF) (http://www.umd.be/HSF) and CADD (https://cadd.gs.washington.edu) (Table 1).

**Figure 1:**
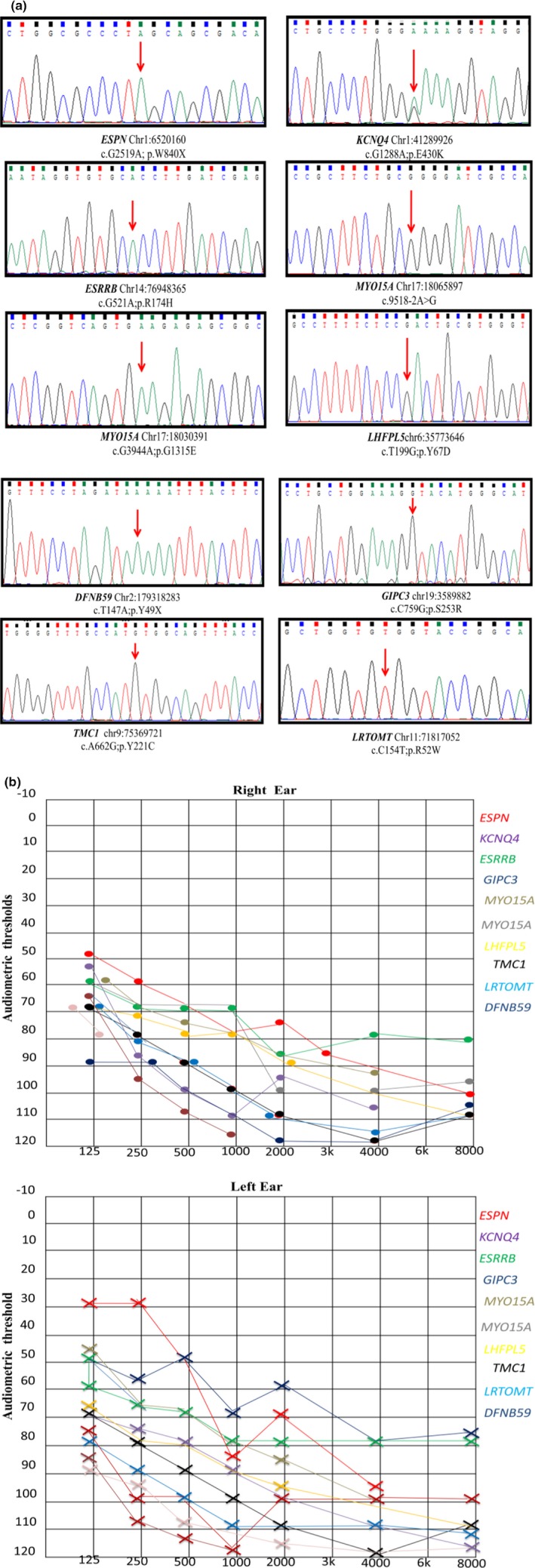
(a) Novel sequence variants identified in *ESPN, KCNQ4, ESRRB, MYO15A, LHFPL5, DFNB59, GIPC3, TMC1,* and *LRTOMT* in our cohort from Pakistan. (b) Audiometry of individuals in our cohort with hearing loss

**Table 1:** Clinical features and genotyping in hearing loss individuals

Subjects	Sex	Age	Gene	Transcript ID	Variant	Allele	Effect on protein	Mutation type	Known/Novel	Variant interpretation prediction scores					ACMG classification
										Polyphen2	PROVEAN	SIFT	Mutation taster	CADD	
1	M	7	*GJB2*	NM_004004.5	c.[71G > A]; [71G > A]	Hom	p.[Trp24*];[Trp24*]	Nonsense	Known	–	–	–	1	36	PVS1
2	M	7	*GJB2*	NM_004004.5	c.[71G > A];[71G > A]	Hom	p.[Trp24*];[Trp24*]	Nonsense	Known	–	–	–	1	36	PVS1
3	M	9	*GJB2*	NM_004004.5	c.[71G > A];c.[231G > A]	Comp Het	p.[Trp24*];p.[Trp77*]	Nonsense	Known	–	–	–	1	36/38	PVS1
4	F	6	*GJB2*	NM_004004.5	c.[370C > T];[370C > T]	Hom	p.[Gln124*];[Gln124*]	Nonsense	Known	–	–		1	34	PVS1
5	F	5	*GJB2*	NM_004004.5	c.[370C > T];[370C > T]	Hom	p.[Gln124*];[Gln124*]	Nonsense	Known	–	–	–	1	34	PVS1
6	M	11	*GJB2*	NM_004004.5	c.[370C > T];[370C > T]	Hom	p.[Gln124*];[Gln124*]	Nonsense	Known	–	–	–	1	34	PVS1
7	F	13	*GJB2*	NM_004004.5	c.[370C > T];[370C > T]	Hom	p.[Gln124*];[Gln124*]	Nonsense	Known	–	–	–	1	34	PVS1
8	M	9	*GJB2*	NM_004004.5	c.[35delG];[35delG]	Hom	p.[(Gly12Valfs*2)];[(Gly12Valfs*2)]	Frameshift	Known	–	–	–	–	–	PVS1
9	F	8	*GJB2*	NM_004004.5	c.[35delG];[35delG]	Hom	p.[(Gly12Valfs*2)];[(Gly12Valfs*2)]	Frameshift	Known	–	–	–	–	–	PVS1
10	F	10	*GJB2*	NM_004004.5	c.[35delG];[35delG]	Hom	p.[(Gly12Valfs*2)];[(Gly12Valfs*2)]	Frameshift	Known	–	–	–	–	–	PVS1
11	F	7	*GJB2*	NM_004004.5	c.[35delG];[35delG]	Hom	p.[(Gly12Valfs*2)];[(Gly12Valfs*2)]	Frameshift	Known	–	–	–	–	–	PVS1
12	M	5	*GJB2*	NM_004004.5	c.[35delG];[35delG]	Hom	p.[(Gly12Valfs*2)];[(Gly12Valfs*2)]	Frameshift	Known	–	–	–	–	–	PVS1
13	M	8	*GJB2*	NM_004004.5	c.[35delG];[35delG]	Hom	p.[(Gly12Valfs*2)];[(Gly12Valfs*2)]	Frameshift	Known	–	–	–	15	–	PVS1
14	M	9	*GJB2*	NM_004004.5	c.[−23 + 1G>A];c.[231G > A]	Comp Het	p.[*N*/A];p.[Trp77*]	Splice site; Frameshift	Known	–	–	–	–	−/38	PVS1
15	M	10	*GJB2*	NM_004004.5	c.[−23 + 1G>A];[−23 + 1G>A]	Hom	p.[*N*/A];[*N*/A]	Splice site	Known	–	–	–	–	–	PVS1
16	M	8	*SLC26A4*	NM_000441.1	c.[679G > C];[679G > C]	Hom	p.[Ala227Pro];[Ala227Pro]	Missense	Known	1	−4.94	0.001	1	30	PM2
17	F	7	*SLC26A4*	NM_000441.1	c.[679G > C];[679G > C]	Hom	p.[Ala227Pro];[Ala227Pro]	Missense	Known	1	−4.94	0.001	1	30	PM2
18	F	7	*SLC26A4*	NM_000441.1	c.[679G > C];[679G > C]	Hom	p.[Ala227Pro];[Ala227Pro]	Missense	Known	1	−4.94	0.001	1	30	PM2
19	M	6	*SLC26A4*	NM_000441.1	c.[716T > A];[716T > A]	Hom	p.[Val239Asp];[Val239Asp]	Missense	Known	0.845	−5.67	0.001	1	29.5	PP2
20	M	7	*SLC26A4*	NM_000441.1	c.[716T > A];[716T > A]	Hom	p.[Val239Asp];[Val239Asp]	Missense	Known	0.845	−5.67.	0.001	1	29.5	PP2
21	M	8	*MYO7A*	NM_000260.3	c.[1258A > T];[1258A > T]	Hom	p.[Lys420*];[Lys420*]	Nonsense	Known	–	–	–	1	41	PVS1
22	M	9	*MYO7A*	NM_000260.3	c.[1258A > T];[1258A > T]	Hom	p.[Lys420*];[Lys420*]	Nonsense	Known	–	–	–	1	41	PVS1
23	F	14	*MYO7A*	NM_000260.3	c.[4838delA];[4838delA]	Hom	p.[Asp1613Valfs*32];[Asp1613Valfs*32]	Frameshift	Known	–	–	–	–	–	PVS1
24	M	13	*MYO15A*	NM_016239.3	c.[9518−2A > G]; [9518−2A > G]	Hom	p.[*N*/A];[*N*/A]	Splice site	Novel	–	–	–	1	19	PVS1
25	F	15	*MYO15A*	NM_016239.3	c.[3944G > A]; [3944G > A]	Hom	p.[Gly1315Glu]; [Gly1315Glu]	Missense	Known	1	−7.7	0.0	1	24.6	PM2
26	M	7	*GRXCR1*	NM_001080479.2	c.[784C > T];[784C > T]	Hom	p.[Arg262*];[Arg262*];	Nonsense	Known	–	–	–	1	50	PVS1
27	M	12	*GRXCR1*	NM_001080479.2	c.[784C > T];[784C > T]	Hom	p.[Arg262*];[Arg262*];	Nonsense	Known	–	–	–	1	50	PVS1
28	M	10	*LHFPL5*	NM_182548.3	c.[199T > G];[199T > G]	Hom	p.[Tyr67Asp];[Tyr67Asp]	Missense	Novel	1	−6.12	0.032	1	27.8	PM2
29	M	8	*LHFPL5*	NM_182548.3	c.[199T > G];[199T > G]	Hom	p.[Tyr67Asp];[Tyr67Asp]	Missense	Novel	1	−6.12	0.032	1	27.8	PM2
30	M	7	*TMPRSS3*	NM_024022.2	c.[727G > A];[727G > A]	Hom	p.[Gly243Arg];[Gly243Arg]	Missense	Known	1	−7.53	0.01	1	35	PM2
31	F	9	*TMPRSS3*	NM_024022.2	c.[1219T > C];[1219T > C]	Hom	p.[Cys407Arg];[Cys407Arg]	Missense	Known	0.997	−3.98	0.122	1	23.3	PM2
32	M	6	*WFS1*	NM_006005.3	c.[2338G > A];[1219T > C]	Hom	p.[Gly780Ser];[Gly780Ser]	Missense	Known	0.896	−1.34	0.060	1	23.7	PM2
33	M	16	*WFS1*	NM_006005.3	c.[2590G > A];[2590G > A]	Hom	p.[Glu864Lys];[Glu864Lys]	Missense	Known	1	−1.68	0.045	1	28.6	PM2
34	M	22	*ESPN*	NM_031475.2	c.[2519G > A];[2519G > A]	Hom	p.[Trp840*];[Trp840*]	Nonsense	Known	–	–	–	1	42	PVS1
35	M	13	*KCNQ4*	NM_004700.3	c.[1288G > A];[1288G > A]	Hom	p.[Glu430Lys];[Glu430Lys]	Missense	Novel	0.956	−0.72	0.376	1	18.19	PP2
36	M	16	*ESRRB*	NM_004452.3	c.[521G > A];[521G > A]	Hom	p.[Arg174His];[Arg174His]	Missense	Novel	0.860	−4.27	0.046	1	27.5	PM2
37	M	10	*DFNB59*	NM_001042702.4	c.[147T > A];[521G > A]	Hom	p.[Tyr49*];[Tyr49*]	Nonsense	Novel	–	–	–	1	35	PVS1
38	M	8	*GIPC3*	NM_133261.2	c.[759C > G];[759C > G]	Hom	p.[Ser253Arg];[Ser253Arg]	Missense	Novel	0.995	−3.87	0.043	0.9994	23.2	PM2
39	F	9	*TMC1*	NM_138691.2	c.[662A > G];[662A > G]	Hom	p.[Tyr221Cys];[Tyr221Cys]	Missense	Novel	1	−6.34	0.045	1	27.3	PM2
40	F	11	*LRTOMT*	NM_001145308.4	c.[154C > T];[154C > T]	Hom	p.[Arg52Trp];[Arg52Trp]	Missense	Known	0.988	−1.79	0.001	1	26.5	PVS1

Note

Abbreviations: Comp. Het, Compound Heterozygous; F, Female; Hom, Homozygous; M, Male.

Furthermore, these novel variants was neither present in the dbSNP (http://www.ncbi.nlm.nih.gov/SNP/),Exome Variant Server (http://evs.gs.washington.edu/EVS/), GnomAD (https://gnomad.broadinstitute.org), Human gene mutation database (HGMD, http://www.hgmd.cf.ac.uk/ac/index.php), 1,000 Genomes (http://www.1000genomes.org/), and Exome aggregation consortium (ExAC) (http://www.exac.broadinstitute.org). Finally, for the interpretation of variants, the American College of Medical Genetics and Genomics (ACMG) 2015 guidelines were used (Table 1) (Richards et al., 2015).

None of the novel variants were present in 200 ethnically matched control individuals confirmed by PCR and Sanger sequencing. The '15 of 40 patients had known variants in *GJB2* (described above), 5  of 40 patients had known variants in *SLC26A4* c.679G > C; p.Ala227Pro*,* 3 of 40 had known variants in *MYO7A* c.1258A > T; p.Lys420*, c.4838delA; p.Asp1613Valfs*32, 2 of 40 had known variants in *GRXCR1* c.784C > T; p.Arg262*, 2 of 40 had known variants in *TMPRSS3* c.727G > A; p.Gly243Arg, c.1219T > C; p.Cys407Arg, 2 of 40 had known variants in *WFS1* c.2338G > A; p.Gly780Ser, c.2590G > A; p.Glu864Lys*,* 1 of 40 had known variant in *ESPN* c.2519G > A; p.Trp840*,1  of 40 had known variant in *LRTMOT* c.154C > T; p.Arg52Trp and were already known to be associated with syndromic or NSHL (Salman et al., 2015; Wang et al., 2017). The *GJB2* variants were the most common variants in our patient cohort. Our study showed that *GJB2* is the most common gene found mutated in our Pakistani cohort which is similar to other cohorts around the world (Salman et al., 2015; Snoeckx et al., 2005; Wang et al., 2017). We found the *GJB2* variants c.35delG; p.Gly12Valfs*2, c.71G > A; p.Trp24* and c. [71G > A]; c. [231G > A]; p. [p.Trp24*]; p. [Trp77*] as the most common, 15 of 40 patients in our cohort however their frequency is variable worldwide (Salman et al., 2015).

In our patient cohort we found 15/40 patients had *GJB2* variants, (5/40) had *SLC26A4,* and (3/40) had *MYO7A* variants responsible for NSHL. We found the incidence of *GJB2* variants is higher compared to other populations. While this would argue for screening for *GJB2* variants in the first place, we found a comparably high incidence for *SLC26A4* and *MYO7A* variants together. Future research could specifically take advantage of using TNGS in the Pakistani cohort to capture a larger proportion of NSHL cases of genetic origin. We expect our study will enhance public awareness toward this hearing loss trouble and genetic counseling significance for the affected families and their members. This study expanded a spectrum of disease causing variants in genes involved in causing hearing loss.

## WEB RESOURCES

The URLs for data presented herein are as follows:

1,000 Genomes: http://www.1000genomes.org/

Exome Variant Server: http://evs.gs.washington.edu/EVS/

ExAC: http://exac.broadinstitue.org/

dbSNP: http://www.ncbi.nlm.nih.gov/SNP/

OMIM: http://www.omim.org/

HGMD: http://www.biobase-international.com/products/hgmd

SIFT: http://sift.jcvi.org/

Polyphen2: http://genetics.bwh.harvard.edu/pph2/

IGV: http://www.broadinstitute.org/igv/

ANNOVAR: http://annovar.openbioinformatics.org/en/latest/

Mutation Taster: http://www.mutationtaster.org/

OMIM: http://www.omim.org/

UCSC Genome Browser: http://genome.ucsc.edu

## CONFLICT OF INTEREST

The authors declare no conflicts of interest.
